# Analyzing Sleep Behavior Using BERT-BiLSTM and Fine-Tuned GPT-2 Sentiment Classification: Comparison Study

**DOI:** 10.2196/70753

**Published:** 2025-11-10

**Authors:** Yihan Deng, Julia van der Meer, Athina Tzovara, Markus Schmidt, Claudio Bassetti, Kerstin Denecke

**Affiliations:** 1 Institute of Computer Science, University of Bern, Neubrückstrasse 10, Bern, Switzerland, +41 316848426; 2 Institute Patient-centred Digital Health, School of Engineering and Computer Science, Bern University of Applied Sciences, Biel/Bienne, Switzerland; 3 Department of Neurology, University Hospital of Bern, Bern, Switzerland; 4 Center for Experimental Neurology - Sleep Wake Epilepsy Center - NeuroTec, Department of Neurology, Inselspital Bern, University Hospital, University of Bern, Bern, Switzerland

**Keywords:** sleep disorder, clinical documentation, free text, sentiment analysis, opinion discrepancy, LLM, prompting, supervised fine-tuning, large language model

## Abstract

**Background:**

The diagnosis of sleep disorders presents a challenging landscape, characterized by the complex nature of their assessment and the often divergent views between objective clinical assessment and subjective patient experience. This study explores the interplay between these perspectives, focusing on the variability of individual perceptions of sleep quality and latency.

**Objective:**

Our primary goal was to investigate the alignment, or lack thereof, between subjective experiences and objective measures in the assessment of sleep disorders.

**Methods:**

To study this, we developed an aspect-based sentiment analysis method for clinical narratives: using large language models (Falcon 40B and Mixtral 8X7B), we are identifying entity groups of 3 aspects related to sleep behavior (day sleepiness, sleep quality, and fatigue). To phrases referring to these aspects, we are assigning sentiment values between 0 and 1 using a BERT-BiLSTM–based approach (accuracy 78%) and a fine-tuned GPT-2 sentiment classifier (accuracy 87%).

**Results:**

In a cohort of 100 patients with complete subjective (Karolinska Sleepiness Scale [KSS]) and objective (Multiple Sleep Latency Test [MSLT]) assessments, approximately 15% exhibited notable discrepancies between perceived and measured levels of daytime sleepiness. A paired-sample *t* test comparing KSS scores to MSLT latencies approached statistical significance (*t*_99_=2.456; *P*=.06), suggesting a potential misalignment between subjective reports and physiological markers. In contrast, the comparison using text-derived sentiment scores revealed a statistically significant divergence (*t*_99_=2.324; *P*=.047), indicating that clinical narratives may more reliably capture discrepancies in sleepiness perception. These results underscore the importance of integrating multiple subjective sources, with an emphasis on narrative free text, in the assessment of domains such as fatigue and daytime sleepiness—where standardized measures may not fully reflect the patient’s lived experience.

**Conclusions:**

Our method has potential in uncovering critical insights into patient self-perception versus clinical evaluations, which enables clinicians to identify patients requiring objective verification of self-reported symptoms.

## Introduction

### Overview

The interplay between sleep quality, daytime fatigue, and daytime sleepiness is critical for understanding and diagnosing sleep disorders. Sleep quality—a multifaceted concept encompassing depth, continuity, and restorative nature of sleep [1]—is commonly assessed through polysomnography (PSG) or questionnaires [2]. Daytime fatigue significantly impacts quality of life [3], while daytime sleepiness reflects the propensity for unintended sleep episodes.

Past studies have shown inconsistencies between subjective experiences and objective measures. For example, Zavecz et al [4] linked self-reported sleep quality to cognitive performance, while O’Donnell et al [5] reported poor alignment between PSG outcomes and patient-reported sleep quality. Similarly, Aurora et al [6] analyzed how well Epworth Sleepiness Scale (ESS) scores correspond to Multiple Sleep Latency Test (MSLT) values. In psychiatric populations, similar dissociations have been reported between perceived and physiologically measured fatigue, as demonstrated by Stanyte et al [7] who found that individuals with anxiety and mood disorders often exhibit mismatched subjective fatigue ratings and objective sleep parameters.

Critically, objective metrics such as sleep onset latency (SOL) and total sleep time (TST) require disorder-contextual interpretation. Valko et al [8] demonstrate that shortened SOL indicates pathological sleep-wake fragmentation in narcolepsy but signifies health in normal sleepers, while subjectively prolonged SOL in insomnia reflects hyperarousal despite normal PSG. Similarly, fatigue—though quantifiable via scales such as the Fatigue Severity Scale (FSS)—lacks consistent PSG correlates across disorders, complicating objectification[9].

Despite these assessments being routinely documented clinically, no study has leveraged real-world clinical records to analyze subjective-objective alignment across disorders.

To address this gap, we propose a novel natural language processing (NLP) framework for modeling subjective-objective alignment through aspect-based sentiment analysis. Our system extracts (1) patient-reported experiences, (2) objective test results (eg, MSLT and PSG), and (3) clinician commentary on alignment or mismatch between the two. This triadic analysis allows for identifying patterns of misperception—for instance, patients who perceive severe sleep onset problems despite normal PSG values.

Unlike prior work that focused on isolated biomarkers such as Apnea-Hypopnea Index (AHI) or ESS scores, our approach uses language models to extract detailed clinical reasoning from documentation. This allows identification of emerging “sleep-wake discrepancy” phenotypes, such as patients with objectively normal sleep but persistently poor subjective reports.

### Sentiment Analysis in Clinical Text

Sentiment analysis in medical and clinical text has received increasing attention over the past decade [10,11]. Clinical documents often contain detailed descriptions of a patient’s health status, including observations, diagnostic findings, and treatment plans. Analyzing this information is important for determining whether patient outcomes are improving or deteriorating, and for assessing the overall impact of a condition on the patient’s well-being. In addition to objective clinical data, patients’ self-reported experiences may also offer useful indicators that complement medical assessments.

Recent developments in pretrained language models, such as those introduced by Devlin et al [12], have enabled the use of BERT-based architectures for sentiment analysis across multiple languages and granularities. In our work, we define sentiment across 5 discrete levels, which facilitates scaling into a normalized range between 0 and 1. Behera et al [13] presented a model combining Word2Vec embeddings with convolutional and recurrent layers (convolutional neural network and long short-term memory), achieving 91.2% accuracy. Their method also includes polarity normalization to improve classification performance. Other established techniques for sentiment and aspect identification in English-language texts include BiLSTM [12,14], TextCNN [15], TextRNN [16], TextRCNN [17,18], and DPCNN [19].

With the emergence of transformer-based models, research has increasingly focused on controlling language model behavior during fine-tuning, inference, and output postprocessing [20]. For example, Ziegler et al [21] applied reinforcement learning (RL) with human feedback to improve output coherence, while Schulman et al [22] proposed the Proximal Policy Optimization (PPO) algorithm for stable policy updates. Pascual et al [23] introduced Keyword2Text, a framework that guides text generation based on semantic similarity and topic constraints. These control strategies can enhance performance in tasks such as sequence labeling and sentiment classification.

Despite these advances, the use of large language models (LLMs) for aspect recognition in German clinical text remains limited. In addition, data privacy regulations restrict the use of commercial cloud-based models in health care environments. To address these challenges, our study investigates the deployment of open-source language models in secure, on-premises settings. We evaluate their performance in aspect recognition using manually annotated German clinical data. Subsequently, we apply sentiment analysis using a fine-tuned BERT model and compare its performance to a German GPT-2 model, which we adapt through supervised learning and reinforcement-based posttraining techniques.

As can be seen in Table 1, prior work has explored sentiment in clinical text [10,11], or compared ESS to MSLT [6], but none have jointly modeled aspect-level sentiment, LLM-based extraction, and alignment between subjective and objective sleep data. Our contributions include:

1. First application of aspect-based sentiment analysis to German clinical sleep data for sleep disorders.
2. Integration of LLM-based aspect extraction (Falcon 40B and Mixtral 8x7B).
3. Five-level sentiment scoring with normalization.
4. Quantified misalignment modeling between patient-reported and physiological sleep data.
5. Identification of misperception profiles at a large scale using real-world clinical records.

**Table 1.** Comparison of related work and contribution of this study.

Study and Method	Domain	Model type	Aspect extraction	Sentiment analysis	Subjective-objective alignment	Sleep focused	Contribution notes
Denecke and Deng [24]	Clinical (general)	Rule-based+ML^a^	Limited	✓	✓	✓	Early sentiment methods in clinical text
Aurora et al [6]	Sleep disorders	Statistical correlation	✓	✓	✓ (sleepiness)	✓	Compared subjective and objective sleepiness (ESS^b^ vs MSLT)^c^
Hermans et al [10]	Sleep misperception	EEG^d^ analysis	✓	✓	✓	✓	Focus on objective EEG markers of misperception
Denecke and Reichenpfader [14]	Clinical NLP^e^	Transformer (RoBERTa)	✓ (limited)	✓	✓	✓	Survey of clinical sentiment tools
Ziegler et al [21]	General NLP	GPT-2+RLHF	✓	✓ (general)	✓	✓	Introduce RL-based^f^ alignment for subjective text
Our study	Sleep disorder	BiLSTM+RoBERTa/GPT-2+RL	✓ (LLM-extracted)^g^	✓ (5-stage scale)	✓ (quantified misalignment)	✓	Joint subjective-objective modeling, LLM-aspect extraction, scoring, feedback mining

a ML: machine learning.

b ESS: Epworth Sleepiness Scale.

c MSLT: Multiple Sleep Latency Test.

d EEG: electroencephalogram.

e NLP: natural language processing.

f RL: reinforcement learning.

g LLM: large language model.

## Methods

### Overview

**Figure 1.**
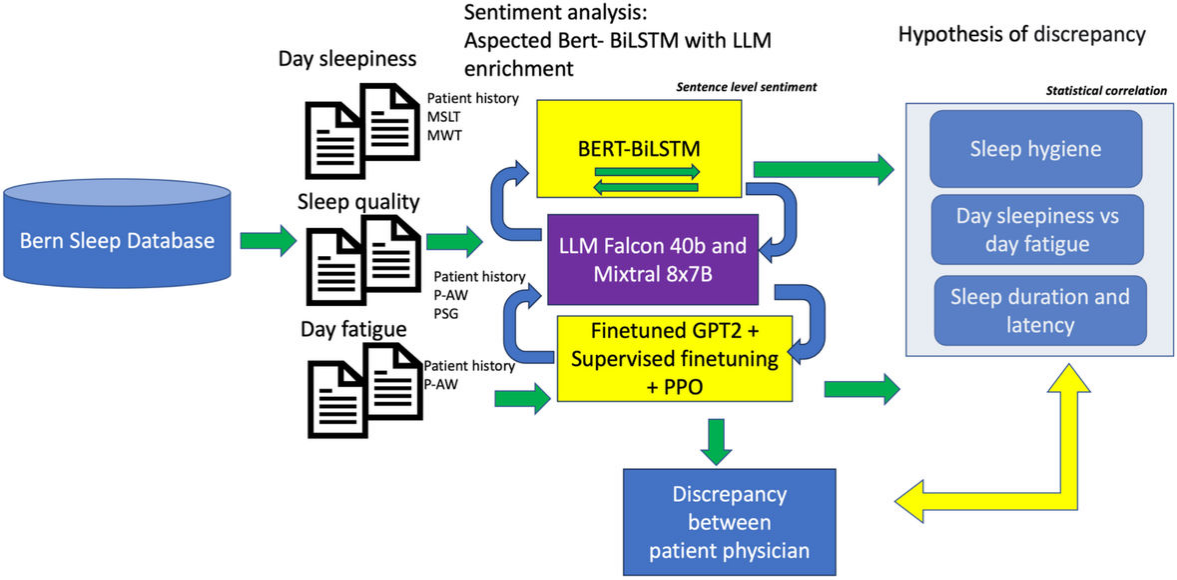
Workflow of sentiment analysis in sleep disorders. LLM: large language model; MSLT: Multiple Sleep Latency Test; MWT: Maintenance of Wakefulness Test; P-AW: Actigraphy Wrist; PPO: Proximal Policy Optimization; PSG: polysomnography.

The developed pipeline for aspect-oriented sentiment analysis is shown in Figure 1. The single steps are described together with the dataset in the following. Our approach involves extracting subjective perceptions of sleep quality, daytime sleepiness, and daily fatigue from the clinical documentation. Specifically, we consider the documented patient history descriptions and the reports related to the PSG examination.

Concurrently, objective benchmarks will be gathered from numerical results in objective sources, including MSLT, MWT (Maintenance of Wakefulness Test), P-AW (Actigraphy Wrist), and parts of the PSG report.

In a first step, entities and phrases referring to our 3 aspects of interest (day sleepiness, sleep quality, and fatigue) are extracted. Once these aspects are identiﬁed, we classify them according to the sentiment expressed for each aspect separately. The next step involves calibrating the objective numerical assessments to match the subjective sentiment scores, allowing for a direct comparison between subjective and objective data. Finally, by evaluating these 2 sets of sentiment values, we statistically analyze the extent of discrepancies in the diagnosis of sleep disorders, providing insights into how patient perceptions align with clinical assessments.

### Dataset

Our dataset originates from inpatient records created at the sleep laboratory of the University Hospital Bern, covering treatments between 2000 and 2021. This collection is part of the Bern Sleep Registry [25]. Its secondary use received approval from the Cantonal Ethics Committee (KEK-Nr. 2022‐00415: “Bern Sleep Registry: the sleep disorder patient cohort of the Inselspital, University Hospital Bern”).

The database comprises German-language coded clinical records of patients diagnosed with sleep disorders. Over 10,000 of these records have been retrospectively and meticulously categorized by physicians specialized in sleep disorders based on the *International Classiﬁcation of Sleep Disorders, Third Edition* (*ICSD-III*). Table 2 shows the types of text documents that are considered from this database for this paper.

**Table 2.** The sentiment analysis mainly focuses on the subjective text (second column), while the objective value extractions are related to test results. Sample sizes reflect prevalence in clinical notes; fatigue records are rarer than sleep quality.

Aspect	Subjective data sources	Information sources and documents	Objective data sources	Statistics
Daytime sleepiness (129 records)	History: Daytime sleepiness, Epworth Sleepiness Scale, MSLT^a^ /MWT^b^ KSS^c^	History, Multiple Sleep Latency Test, Maintenance of Wakefulness Test	MSLT and MWT: eg, “In summary, a mild daytime sleepiness can be objectiﬁed,” vigilance test results	36 (28%) of records show SOL-KSS^d^ discrepancy ESS^e^ >10 correlates with MSLT latency <8 minutes (*r*=–0.72) "Mild daytime sleepiness" documented in 58 (63%) of 92 cases with MSLT 5-8 min
Fatigue exhaustion depressive symptoms (450 records)	History: Fatigue, Fatigue Severity Scale, BDI-II^f^	Hist^g^, P-AW^h^	Increased inactivity component	FSS^i^ >5 correlates with BDI-II >14 (r=0.68) 189 (42%) show activity reduction "Increased inactivity" noted in 112 (89%) of 126 severe fatigue cases
Sleep quality (3815 records)	Hist: Sleep quality, Insomnia PSG^j^ Report: Estimated sleep onset Latency and sleep duration	Hist, PSG, P-AW	PSG Report: Objective sleep onset Latency and sleep duration, sleep efficiency (¡ 80%: reduced). Actigraphy Report: Actimetric sleep efficiency	2557 (67%) underestimate TST^k^ (mean diff: –73 min) SE <80% in 82%^l^ of insomnia diagnosis "Reduced efficiency" documented in 94%^m^ of SE<75% cases

a MSLT: Multiple Sleep Latency Test.

b MWT: Maintenance of Wakefulness Test.

c KSS: Karolinska Sleepiness Scale.

d SOL-KSS: Sleep Onset Latency Karolinska Sleepiness Scale

e ESS: Epworth Sleepiness Scale.

f BDI-II: Beck Depression Inventory II.

g Hist: patient history.

h P-AW: Actigraphy Wrist.

i FSS: Fatigue Severity Scale.

j PSG: polysomnography.

k TST: total sleep time.

l Absolute number not available; based on weighted insomnia subset

m Absolute counts unavailable due to conditional subgroup definitions.

For various validations, we have prepared the following manually annotated datasets to validate and ﬁne-tune the pretrained model:

1. Aspect extraction benchmark: 150 sentences manually labeled to validate entity and aspect extraction performance.
2. Sentiment analysis corpus: 2000 sentences annotated on a 5-point sentiment scale using on-premises LLMs (Falcon 40B [26,27] and Mixtral 8x7B [28]), with human-in-the-loop correction.

The 2000 sentences were sampled from clinical documents belonging to 100 unique patients, spanning multiple sleep disorder categories. These sentences were drawn proportionally from patient histories, diagnostic summaries, and follow-up reports to ensure a representative mix across conditions and report types. Stratified sampling was used to balance diagnoses, age groups, and sentiment distribution. To avoid patient-level bias, no more than 10 sentences per patient were included in the annotated set.

To ensure robust analysis of subjective-objective alignment in daytime sleepiness, we included only those patients who had complete paired data for the following instruments: Karolinska Sleepiness Scale (KSS)—subjective measure, MSLT—objective physiological measure, and MWT—behavioral sleepiness measure. This resulted in a final cohort of 100 patients with complete records across all 3 modalities, allowing for valid computation of discrepancy and misperception scores. For other dimensions, such as fatigue and sleep quality, which currently lack standardized objective clinical benchmarks, we retained a broader set of records (eg, 3815 entries for sleep quality) to support exploratory sentiment-based analysis.

### Aspect and Sentiment Distribution

To analyze sentiment by aspect, we annotated 50 representative sentences per aspect totaling 150 sentences. Table 3 summarizes the frequency of extracted entity groups by category, while Table 4 presents the sentiment annotations and related statistics.

**Table 3.** Aspect benchmark statistics regarding sleep quality, daytime sleepiness, fatigue, and the entire corpus.

Aspects	List of entity groups	Entity count
Sleep quality (50 sentences)	Sleep quality, Latency, Duration, Estimation	75
Daytime sleepiness (50 sentences)	Day sleepiness, Latency, PVT (Vigilance test), ESS^a^/KSS^b^	35
Fatigue (50 sentences)	Fatigue Severity Scale, Measurements BDI-II^c^	77
Entire benchmark (150 sentences)	Symptoms, Diseases, Complaints, Feedback, Measurements	119

a ESS: Epworth Sleepiness Scale.

b KSS: Karolinska Sleepiness Scale.

c BDI-II: Beck Depression Inventory II.

**Table 4.** Sentiment statistics regarding sleep quality, daytime sleepiness, fatigue, and the entire corpus.

Sentiment class	Count	Percentage	Average sentence length	Lexical characteristics
Very Negative	180	9%	22.4 (5.8) words	Sleep quality: "severe insomnia," "non-restorative sleep," "fragmented sleep all night" Fatigue: "debilitating exhaustion," "unable to function" Sleepiness: "dangerous sleep attacks," "uncontrollable drowsiness"
Negative	680	34%	18.6 (4.2) words	Sleep quality: "frequent awakenings," "prolonged sleep latency," "restless sleep" Fatigue: "persistent tiredness," "low energy throughout day" Sleepiness: "excessive daytime sleepiness," "struggling to stay awake"
Neutral	920	46%	14.2 (3.1) words	Sleep quality: "TST 6.2 hours," "sleep efficiency 82%," "PSG: 4 REM cycles" Fatigue: "reports moderate fatigue," "FSS score: 4.2" Sleepiness: "ESS: 12," "MSLT mean latency: 8.3 min"
Positive	180	9%	16.8 (3.9) words	Sleep quality: "improved sleep continuity," "satisfactory sleep duration" Fatigue: "reduced fatigue levels," "manageable tiredness" Sleepiness: "mild daytime sleepiness," "occasional drowsiness"
Very Positive	40	2%	19.3 (4.7) words	Sleep quality: "excellent sleep quality," "fully restorative sleep" Fatigue: "complete resolution of fatigue," "sustained energy" Sleepiness: "full alertness," "no daytime sleepiness"
Total	2000	100%	16.8 (4.6) words	—

a TST: total sleep time.

b PSG: polysomnography

c REM: rapid eye movement.

### Mathematical Description of Aspect-Based Sentiment Analysis for Sleep Disorder

In the following, we define the dataset and tasks considered in this paper.

Dataset Definition

Let the dataset be defined as:

*D*={(*x_i_*, *A_i_*, *y_i_*)|=1,...,*N*

Where:

- *x_i_* is the tokenized and embedded representation of sentence *i*, obtained via BERT or RoBERTa (shape: *T×d*, where *T*=tokens, *d*=embedding dimension).
- *A_i_* is the set of extracted aspect terms from *x*_*i*_ using LLMs (eg, Falcon 40B and Mixtral 8x7B).
- *y_i_* is the discrete sentiment label ∈ {0, 1, 2, 3, 4} representing 5-stage sentiment levels.
- The normalized score is:
  $s_{i}=\frac{y_{i}}{4}$So *S_i_*∈[0,1]

Aspect Extraction via LLMs

For each input sentence *x_i_* and aspect category, for example, *c* ∈ C_Categories, we define a prompt *P*_*c*_ and extract:

*a_i_*^(^*^c^*^)=*LLM* (*P_c_,x_i_*)^ for each aspect category c

The union of these yields the aspect set:

*A_i_*=*U_c_a_i_*^(^*^c^*^)^

To align with canonical medical terms, compute cosine similarity:

sim(e, c) = cosine(embedding(e), embedding(c))

Accept the match if sim≥threshold

Sentiment Classification (BiLSTM+Self-Attention)

1. Input embeddings
  Each *x*_*i*_*R* is passed to a bidirectional LSTM:
*h*_*i*_ *=BiLSTM (x*_*i*_ *)∈R* ^*T×h*^
2. Sequential feature aggregation (concatenation or pooling):
*f*_*i*_ *=Self – Attention (h*_*i*_ *)∈R* ^*h′*^
3. Classification layer (with softmax over 5 sentiment classes): Using a linear layer with parameters *W*, *b*:
  $p_{i}=softmax\left(W\times f_{i}+b\right)$ with ${\hat{y}}_{i}=argmax\left(p_{i}\right)$
  Where:

- *W*∈*R*^5×*h*′^, *b*∈*R*^5^are learnable parameters,
- *p_i_*∈*R*^5^ is the predicted class distribution.

Training Objective (Supervised Fine-Tuning: L1)

Use categorical cross-entropy for supervision:


$$L_{CE}=\frac{-1}{N}\sum_{i=1}^{N}\sum_{k=1}^{5}1\left[y_{i}=k\right]∙log(p_{i},k)$$

Sentiment Control via GPT-2 (L2: Reinforcement Learning)[29]

After initial fine-tuning, a GPT-2 model is trained to modify or generate text while controlling for sentiment class y_i_

Logit Biasing /Reward Shaping: to


$$L_{RL}=E_{\hat{x_{i}}\pi_{\theta }}\left[r\left(\hat{x_{i}},y_{i}\right)\right]$$

Where $r\left(\hat{x_{i}},y_{i}\right)$ is a reward signal based on:

- Keyword inclusion (aspect control),
- Matching predicted sentiment from a fixed classifier with *y_i_*

The full objective (SFT+RL) becomes:

*L_total_*=*L_CE_*+λ·*L_RL_*

where λ∈[0,1] is a hyperparameter controlling the influence of reinforcement-based alignment.

Normalization of Subjective and Objective Measures

Min-max normalization for raw clinical scores *r* over scale [*S_min_*, *S_max_*]:


$$s=\frac{S_{max}-r}{S_{max}-S_{min}}$$

Examples:

- Epworth Sleepiness Scale (0‐24):

$s=\frac{24-r}{24}$

- Fatigue Severity Scale (1-7):$s=\frac{7-r}{6}$
- Beck Depression Inventory II (0‐63)

$s=\frac{63-r}{63}$

- Karolinska Sleepiness Scale (KSS) (1-9):

$s=\frac{9-KSS}{9-1}$

### Aspect Extraction From Diagnostic Text

For aspect extraction, we define 4 distinct groups of clinical entities based on their frequency and contextual relevance, with the aim of analyzing discrepancies between subjective reports and objective clinical findings (see Table 2). While aggregating sentiment scores can offer a general view of polarity across different textual spans (eg, sentences or paragraphs), they are insufficient for capturing detailed sentiment variations related to specific medical aspects such as symptoms, complaints, diagnoses, or co-occurring conditions. To address this limitation, we implemented a fine-grained entity extraction approach that combines open-source LLMs with standard clinical entity recognition techniques.

Specifically, we use 2 self-hosted models: Falcon 40B [27] and Mixtral 8x7B [30]. Falcon 40B is a dense, decoder-only language model designed for a broad range of NLP tasks. Mixtral 8x7B, by contrast, follows a sparse mixture-of-experts architecture, activating 2 expert modules per token, thereby achieving a favorable balance between performance and computational efficiency. Despite having 46.7 billion parameters in total, only a subset is used at each step, making it suitable for resource-constrained environments. We evaluated Falcon 40B as a baseline but used Mixtral 8x7B for all final extractions reported here.

Aspect-level sentiment scoring is achieved by linking the extracted entities to their corresponding sentiment values within a given sentence. This allows us to compute sentiment scores that are specific to individual clinical aspects. The entity extraction process is carried out in 2 stages: (Stage 1) Aspect identification using prompt-based querying with the locally hosted Mixtral 8x7B model. Separate prompts are constructed for each category—symptoms, complaints, diagnoses, and patient feedback—to ensure focused and interpretable extraction.

To extract patient feedback entities, we design targeted natural language prompts structured to guide the model toward recognizing relevant subjective expressions. Below is a representative prompt used with the Falcon 40B model:

#### Prompt

You are a helpful clinical entity extractor. Given the following medical note, please extract any patient feedback or evaluative statements related to their condition, treatment experience, or overall well-being.

Text: “[Insert clinical sentence here]”

Output format: [feedback_1, feedback_2, ...]

The given prompt for entity extraction from clinical patient data can be efficiently reused by systematically substituting the target category in the text. By iteratively inserting each category (eg, “symptoms,” “diseases,” “complaints,” and “feedback”) into the prompt, the same prompt structure can be applied multiple times to extract entities for one category at a time. This approach allows looping over the list of aspect categories and generating a valid JSON object for each without needing multiple distinct prompts. It ensures structured and repeatable extraction across all defined aspects.

To improve the identification of measurement-related entities in German clinical texts, which are often missed by LLMs due to domain-specific phrasing in German clinical texts, we apply a semantic similarity approach based on sentence embeddings. This method is more robust to morphological variation, word order, and paraphrasing than traditional string metrics such as Levenshtein distance.

We used German-specific sentence embeddings generated by the sentence-transformers library with the multilingual model distiluse-base-multilingual-cased-v1, which supports high-quality semantic representations of German phrases.

#### Matching Procedure

To identify and align relevant sleep-related entities from clinical narratives, we implemented a multistep matching procedure combining lexical, semantic, and synonym-based techniques, as detailed below:

1. Entity candidate generation: Candidate phrases were extracted from clinical documents using regex-based pattern matching and contextual heuristics (eg, token windows around sleep-related terms).
2. Embedding computation: Sentence embeddings were computed for each candidate phrase and compared to embeddings of canonical terms from our curated sleep measurement lexicon.
3. Similarity scoring: Cosine similarity was used to measure semantic closeness between candidate and canonical embeddings. A similarity threshold of 0.83 was used to determine a valid match based on manual validation (see Table 5).
4. Synonym expansion: A manually defined dictionary of common medical synonyms (eg, “Schlafqualität” ≈ “Qualität des Schlafes”) was integrated into the matching logic to further boost recall.

**Table 5.** Embedding-based semantic matching for German sleep disorder terms.

Canonical term	Variant detected in text	Cosine similarity	Matched?	English translation
Schlafqualität	Qualität des Schlafes	0.86	True	Sleep quality
Schlaflatenz	Zeit bis zum Einschlafen	0.85	True	Time to fall asleep
REM-Schlafanteil^a^	REM Schlafphasen Dauer	0.81	False	Duration of REM sleep phases

a REM: rapid eye movement.

### Objective Measure Calibration

To enable comparability across heterogeneous clinical measurements and support sentiment interpretation, we apply min-max normalization to both objective and subjective metrics. This ensures all scores lie within the interval [0, 1], where 0 indicates maximal symptom burden and 1 indicates no impairment. This transformation provides a unified scale for downstream sentiment mapping.

For objective physiological measures such as PSG-recorded sleep latency, we applied the following normalization: Normalized Latency Score=1–(Raw Value/Max Latency) where the clinically derived maximum latency was set to 120 minutes, in accordance with standard clinical PSG protocols. This formulation ensures shorter latency (indicative of better sleep initiation) corresponds to higher normalized scores.

#### Subjective scale normalization

For subjective clinical questionnaires (see Table 6), we used min-max normalization based on each scale’s full range. The transformation ensures that higher symptom burden maps to lower normalized values:

Normalized Score=(Max Score – Raw Score) /(Max Score – Min Score)

**Table 6.** Normalization mappings between the objective score.

Instrument	Score range	Normalization formula
Karolinska Sleepiness Scale (KSS)	1‐9	(9 – Raw Score) / (9 – 1)
Epworth Sleepiness Scale (ESS)	0‐24	(24 – Raw Score) / (24 – 0)
Fatigue Severity Scale (FSS)	1‐7	(7 – Raw Score) / (7 – 1)
Beck Depression Inventory II (BDI-II)	0‐63	(63 – Raw Score) / (63 – 0)

This ensures that 0 corresponds to the maximum raw score (most negative sentiment), and 1 corresponds to the minimum raw score (most positive sentiment).

### Architecture for Clinical Sentiment Analysis

**Figure 2.**
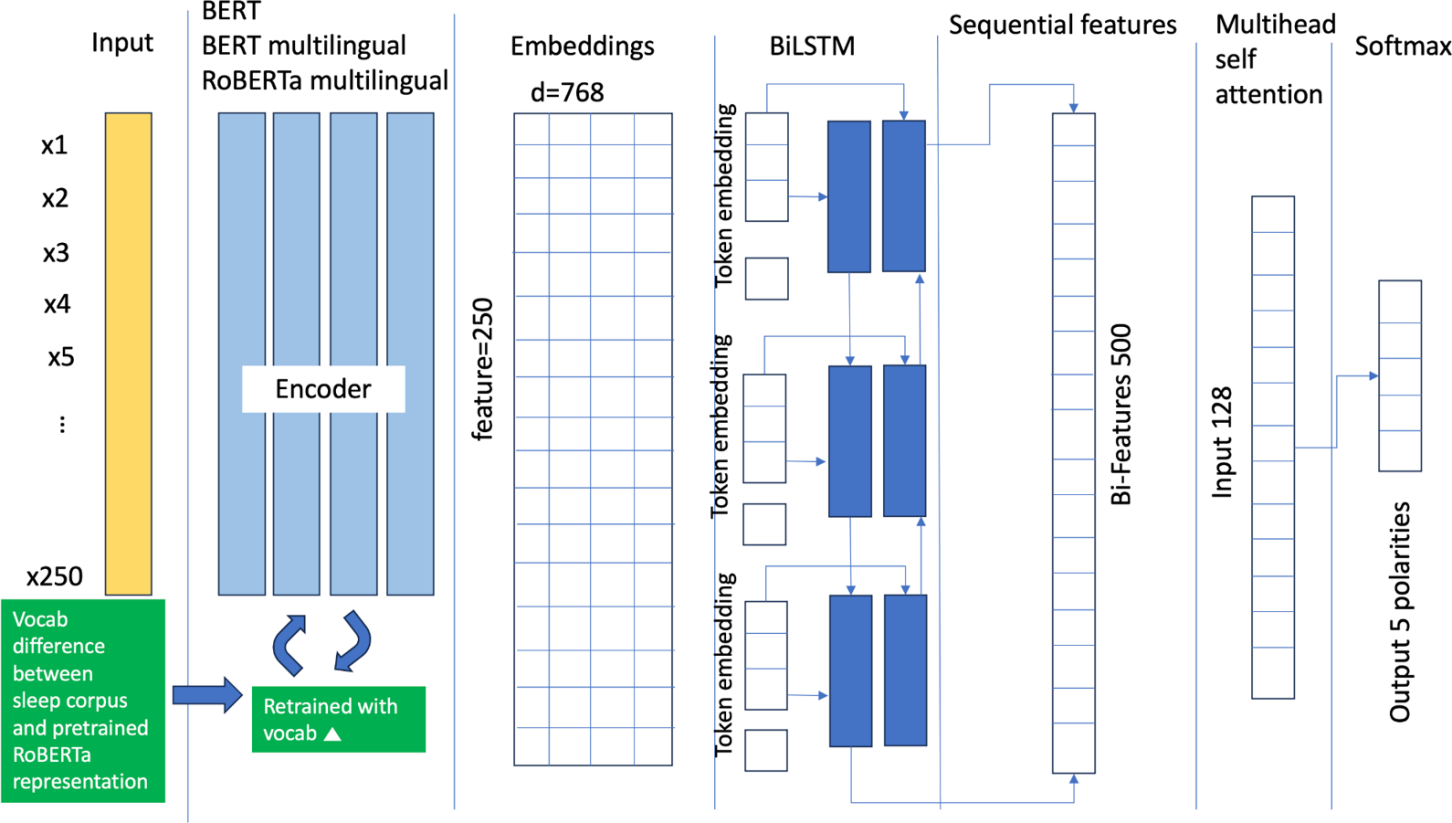
BiLSTM based on BERT language modeling for clinical sentiment analysis.

We deploy a BERT-based BiLSTM architecture to perform sentence-level sentiment classification across 5 polarity levels (see Figure 2). The model ingests token sequences up to 250 tokens, with BERT embeddings (768 dimensions) [31] providing context-aware representations.

We fine-tune both multilingual BERT and XLM-RoBERTa [32] on a corpus of 1200 German clinical sentences annotated with 5-stage sentiment labels, derived from Mixtral 8X7B prompting and manual correction (interannotator agreement >89%).

Negated expressions in German are normalized into unified tokens prior to BERT encoding to stabilize polarity signals. The resulting embeddings are processed by a BiLSTM layer followed by sequential feature aggregation. The output logits are mapped to 5 sentiment classes, which are rescaled to a [0, 1] range:

0 (very negative), 1 (negative), 2 (neutral), 3 (positive), 4 (very positive)

The model is trained using a 60/20/20 split across training (1200), validation (400), and test (400) sets.

#### Feature Fusion and Context Learning

The architecture incorporates multiple contextual sources:

*F_Mask_*: local contextual features via dynamic masking

*F_weighting_*: Dynamically weighted local embeddings

*F_disorder_*: global BiLSTM features

The 5 stages of sentiment resulting from with or without BERT alignment and ﬁne-tuning will be evaluated on these benchmarks.

#### Feature Ensemble and Context Learning for BERT BiLSTM

During the uniﬁed contextual feature assembling process for sleep disorder topics, both local and global contextual features derived from the contextual feature dynamic mask technique, the dynamic weighting mechanism, and the BiLSTM layers are integrated. In addition, features enriched by LLMs are incorporated. Speciﬁcally, the learned local and global contextual features are concatenated to produce a uniﬁed contextual feature output. This procedure is mathematically represented as follows:


$$F_{ensemble}=F_{mask}^{l}⨁F_{weighting}^{l}⨁F_{disorder_{aspect}}^{g}$$

In this equation, $F_{mask}^{L}$ represents the set of local contextual embedding vectors computed by the dynamic mask technique, while $F_{weighting}^{L}$ denotes the set of local contextual embedding vectors examined through the dynamic weighting mechanism. *F*_*disorder*_ aspect refers to the set of global contextual embedding vectors captured via BiLSTM layers. The operator indicates the concatenation of these feature sets. These fused features are further processed through BiLSTM to capture sequential dependencies and then transmitted into Feedforward Network with Softmax to perform ﬁne-grained aspect-based sentiment classiﬁcation.

#### Network Conﬁguration and Hyperparameters for BERT BiLSTM

The hyperparameter Table 7 outlines the conﬁguration of the network, which operates in 2 main stages. In the ﬁrst stage, aspect recognition is performed using the initial set of network parameters. The recognized aspects are then incorporated into the second stage through feature fusion, where they are linearly combined and pooled together with position features. This fused feature set is subsequently applied and jointly optimized within the ﬁne-tuned RoBERTa model’s loss function. The optimization is carried out using a 5-stage polarity classiﬁcation, ensuring that both aspect-level insights and positional information are effectively used for enhanced performance.

**Table 7.** Model hyperparameters for ﬁne-tuning of BERT and RoBERTa BiLSTM multilingual.

Parameter	Value
Embedding dimension	768
Transformer encoder	12
Attention head	12
Optimizer	Adam^a^
Learning rate	5e-5
Epoch	50
Dropout rate	0.2
Batch size	32

a Adam: adaptive moment estimation is a stochastic optimization algorithm.

#### Finetuned German GPT-2 With Supervised Fine-Tuning and Logit Modiﬁcation

**Figure 3.**
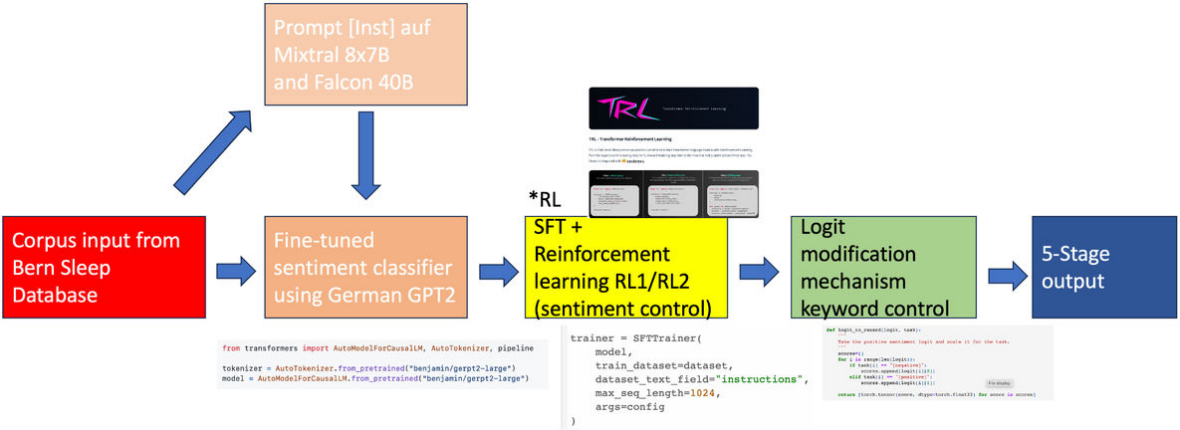
Fine-tuned German GPT-2 followed by supervised ﬁne-tuning, reinforcement learning, and logit modiﬁcation. *RL indicates the steps of reinforcement learning RL1 and RL2. RL: reinforcement learning; SFT: supervised fine-tuning.

In addition to our BERT-BiLSTM sentiment classification baseline, we fine-tune a German GPT-2 model [26,33,34] to generate text reflecting 5 clinically relevant stages of sentiment in sleep disorder narratives. As illustrated in Figure 3, our 2-stage fine-tuning approach involves both supervised learning (L1) and reinforcement learning (L2) to align the model’s generations with targeted sentiment levels. In the first stage (L1), we perform supervised fine-tuning (SFT) using the Hugging Face Transformer Reinforcement Learning (TRL) framework [28] on a corpus of 1200 annotated German clinical texts (Table 8), each labeled with 1 of 5 sentiment classes: strong negative, minor negative, neutral, minor positive, and strong positive. The SFT phase runs for 3 epochs with a learning rate of 5e-5, a batch size of 16, and a maximum sequence length of 1024 tokens, using standard language modeling loss to adapt the pretrained GPT-2 model to the clinical sleep domain and sentiment-specific instruction format. Further details regarding the implementation are available in the GitHub repository [35].

**Table 8.** Model hyperparameters for GPT-2 supervised fine-tuning.

Parameter	Value
GPT-2 sentiment classiﬁer learning rate	2e-5
GPT-2 sentiment classiﬁer batch size	16
SFT trainer learning rate	5e-5
SFT training epochs	3
SFT maximum sequence length	1024
RL^a^ generation kwargs top-k	3
RL generation kwargs top-p	0.5
Maximum new tokens	100
RL PPO^b^ mini-batch size	16
RL PPO conﬁguration steps	41,000

a RL: reinforcement learning.

b PPO: Proximal Policy Optimization.

The second stage (L2) consists of RL via PPO [33], using the sentiment classifier as a reward model. Here, the model generates responses conditioned on prompts and randomly sampled target sentiment tokens. The classifier evaluates each generation and assigns a reward signal based on the logit of the target sentiment class, encouraging the model to reinforce sentiment-accurate responses. This process is iteratively optimized over 41,000 PPO steps, using a PPO mini-batch size of 16, and generation hyperparameters including top-k=3, top-p=0.5, and a maximum of 100 new tokens per sample.

We designate these PPO training phases as RL1 and RL2, which are now clearly labeled in Figures1  3 *RL. Each RL phase refines the generator’s ability to follow sentiment conditioning with increasing precision. This logit-based reward shaping strategy ensures granular control over sentiment realization in the output. Our evaluation metric during PPO optimization is the accuracy of generated sentiment, as classified by the same frozen classifier used for reward computation. The combination of supervised preadaptation and reward-driven fine-tuning allows our GPT-2 model to generate sentiment-aligned narratives that preserve clinical plausibility while reflecting emotional tone variations essential for downstream affective or patient-centered NLP tasks.

#### Derived Subjective Sleepiness Scoring and Comparison to Objective Measures

##### Subjective Sleepiness Approach

To robustly assess subjective sleepiness, we combined inputs from the KSS and clinician-documented descriptions to produce a harmonized subjective score. KSS ratings were first converted to a 5-stage ordinal scale (0‐4), with thresholds mapped as follows: 1‐2=4 (very severe sleepiness),

3‐4=3, 5‐6=2, 7‐8=1, 9=0 (no sleepiness).

This reverse scoring reflects the interpretation that lower KSS values indicate greater momentary sleepiness.

In parallel, clinical text segments were processed using a sentiment analysis pipeline based on a fine-tuned GPT-2 model trained with RL to optimize accuracy across 5 sentiment stages. This model provided a text-derived sleepiness score on the same 0‐4 scale, where 0 indicated no subjective complaints and 4 indicated severe functional impairment due to sleepiness.

##### Objective Sleepiness Derivation

The MSLT was used to obtain an objective index of daytime sleepiness. Since shorter latencies reflect greater sleepiness, raw MSLT values were inverted and scaled onto a 0‐4 range: objective score = ((20 – MSLT latency) / 20)×4. This allowed direct comparison of subjective and objective sleepiness on the same scale.

##### Paired Comparison and Findings

To evaluate the degree of alignment between subjective and objective sleepiness, we performed a paired-sample *t* test. For each patient, we computed the difference between the harmonized KSS sleepiness stage and GPT-2-RL–derived text score and the corresponding objective sleepiness score derived from MSLT latency.

The paired-sample *t* test evaluates whether the mean difference between the subjective and objective scores across all patients significantly differs from zero. The test statistic is calculated using:


$$t=\frac{\bar{d}}{S_{d}/\sqrt{n}}$$

where *d* is the mean of the differences between paired observations:


$$\bar{d}=\frac{1}{n}\sum_{i=1}^{n}\left(x_{i}-y_{i}\right)$$

*S_d_* is the standard deviation of the differences:


$$S_{d}=\sqrt{\frac{1}{n-1}\sum_{i=1}^{n}{\left(d_{i}-\bar{d}\right)}^{2}}$$

*n* is the number of paired samples, *x_i_* is the subjective score for patient *i*, and *y_i_* is the objective score for patient *i*.

### Ethical Considerations

Ethical approval for this study was obtained from the Kantonale Ethik Kommission Bern (Cantonal Ethics Committee Bern) for multiple project components: Project part 1: SNS Project (2000-2016), BASEC-ID 2016-00409 and Project part 2: Bern Sleep Registry (“The sleep disorder patient cohort of the Inselspital, University Hospital Bern*”*), KEK-Nr. 2022-00415.

The secondary use of data from the Bern Sleep Registry was also approved by the Cantonal Ethics Committee. All data were handled in accordance with institutional and Swiss data protection regulations. Informed consent was obtained as required for each project component, and all participants were informed of their ability to opt out of data use. All data were fully de-identified prior to analysis to protect participant privacy and ensure compliance with applicable data protection standards.

## Results

### Overview

This section presents the results of our aspect-based sentiment analysis pipeline, including [1] 5-stage sentiment classification using transformer-based models, and [2] LLM-based extraction of clinical entity aspects. The analyses were applied to a dataset of sleep-related medical texts and further supported by clinical insights into subjective-objective misperception.

### Evaluation of the Aspect-Based Sentiment Analysis

Table 9 summarizes the performance of several models for 5-stage sentiment classification, evaluated using standard multiclass metrics. Initial models such as BERT and RoBERTa achieved moderate performance (accuracy 61% and 69%, respectively). Incorporating a BiLSTM layer improved performance to 78%. Further gains were achieved with a fine-tuned GPT-2 model. SFT alone yielded 81% accuracy, and RL with logit-space modulation increased it to 87%. These results demonstrate the effectiveness of multistage alignment in modeling sentiment nuances. Metrics reported in Table 9 are macro-averaged across 5 sentiment classes, based on an 80/20 stratified test split.

**Table 9.** Performance of sentiment classiﬁcation of semiautomatically annotated 5-stage sentiment. Training: 1200 sentences (60%), Validation: 400 (20%), Test: 400 (20%) for hyperparameter optimization.

Sentiment task	Accuracy	AUC-ROC^a^	Precision (macro)	Recall (macro)	*F*_1_-score (macro)
BERT 5-stage	0.61	0.76	0.62	0.60	0.61
RoBERTa 5-stage	0.69	0.84	0.70	0.68	0.69
RoBERTa+BiLSTM	0.78	0.90	0.79	0.77	0.78
GPT-2+SFT^b^	0.81	0.93	0.82	0.81	0.81
GPT-2+SFT+RL1^c^+ logit mod	0.85	0.95	0.86	0.85	0.85
GPT-2+SFT+RL2+logit mod	0.87	0.96	0.88	0.87	0.87

a AUC-ROC: area under the receiver operating characteristic curve.

b SFT: supervised ﬁne-tuning.

c RL: reinforcement learning.

All metrics were computed using macro-averaging, giving equal weight to all sentiment classes. The AUC-ROC (area under the receiver operating characteristic curve) values were obtained using a one-vs-rest strategy, macro-averaged across 5 classes. These approaches ensure that both frequent and rare sentiment classes are equally represented in performance assessment.

**Figure 4.**
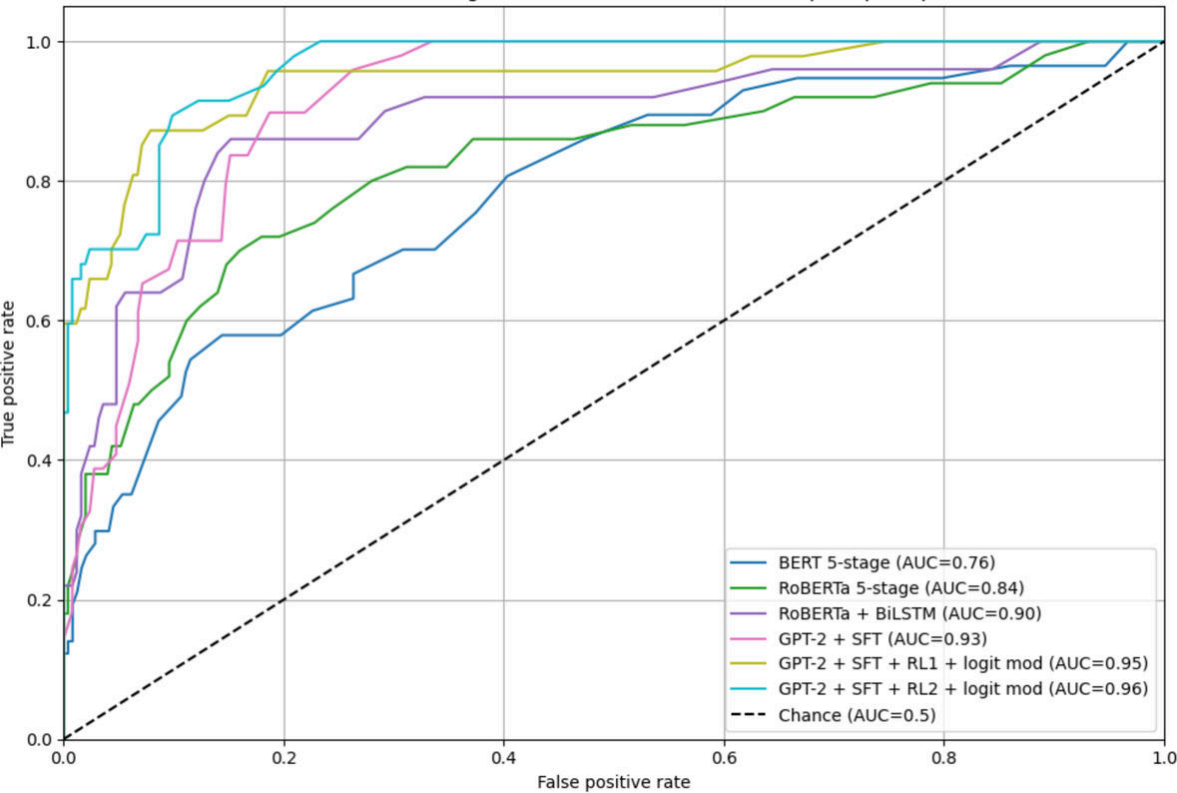
AUC-ROC (area under the receiver operating characteristic curves) for 5-class sentiment classification across transformer-based models. SFT: supervised fine-tuning.

Figure 4 visualizes the ROC curves of the models. GPT-2 variants with SFT+RL training consistently outperformed the baselines, reflecting enhanced class separability through reinforcement learning and logit regularization.

The ROC-AUC curves illustrate the one-vs-rest performance of 6 models on the 5-stage sentiment classification task using clinical text from sleep disorder records. All models perform substantially above the chance level (AUC=0.5), with performance steadily improving from BERT (AUC=0.76) to RoBERTa (AUC=0.84), and further to RoBERTa+BiLSTM (AUC=0.90). Fine-tuned GPT-2 (SFT) achieves strong performance (AUC=0.93), which is further enhanced by reinforcement learning (RL1 and RL2) and logit modification, reaching up to AUC=0.96. The stair-step shapes reflect the small test set (400 samples), but the consistent trend shows that reinforcement learning significantly boosts the model’s ability to distinguish fine-grained sentiment levels, supporting its potential for clinical decision support in contexts such as misperception of sleepiness.

### Aspect Extraction Through LLM Falcon 40B and Mixtral 8X7B

Clinical entity extraction was performed using prompting strategies applied to Falcon 40B and Mixtral 8X7B models. Table 10 compares their performance on 4 annotated categories: Symptoms, Diseases, Complaints, and Feedback. Mixtral 8X7B showed strong performance with macro *F*_1_ of 0.8490, outperforming Falcon 40B (macro *F*_1_ of 0.7265).

**Table 10.** Performance of the aspect extraction and benchmarks: 150 manually labeled sentiment aspects.

Performance of aspect extraction	Precision	Recall	Micro *F*_1_	AUC-ROC^a^
Falcon 40B instruct entity extraction				
Symptoms	0.8157	0.7963	0.8054	0.903
Diseases	0.8321	0.8485	0.8397	0.920
Complaints	0.7829	0.7134	0.7466	0.873
Feedback	0.5416	0.4896	0.5142	0.757
Marco *F*_1_	—^b^	—	0.7265	—
Mixtral 8X7B Instruct v0.1 entity extraction				
Symptoms	0.9175	0.9114	0.9054	0.953
Diseases	0.8812	0.8804	0.8797	0.940
Complaints	0.8411	0.8338	0.8266	0.913
Feedback	0.8616	0.8028	0.8312	0.903
Marco *F*_1_	—	—	0.8490	—

a AUC-ROC: area under the receiver operating characteristic curves.

b Not available.

Since Mixtral 8X7B consistently outperformed Falcon 40B across all evaluated categories, we applied Mixtral 8X7B for all entity extraction tasks in our sentiment analysis pipeline. Beyond the benchmark evaluation, the model extracted a total of 873 unique symptom terms, along with 59 distinct diseases, 432 complaints, and 224 feedback-related entities across the full clinical corpus.

#### Sentiment Distribution Patterns

Currently, there are no established objective ground truth standards for evaluating misperception in insomnia, and for disorders such as hypersomnia or sleep-disordered breathing, further clinical validation is required. Thus, our focus was placed on daytime sleepiness, where both subjective (KSS) and objective (MSLT and MWT) data were consistently available.

The KSS and the ESS both assess subjective sleepiness but differ in temporal focus: KSS captures momentary state-level sleepiness, while ESS reflects trait-level sleepiness across habitual situations. Because KSS is time-specific, it aligns more directly with objective measures such as MSLT latency.

In this study, we focus exclusively on KSS—with or without clinical text—to assess momentary misperception in daytime sleepiness. This approach avoids confounding from chronic perception scales such as ESS and enables precise modeling of state-dependent discrepancies.

**Figure 5.**
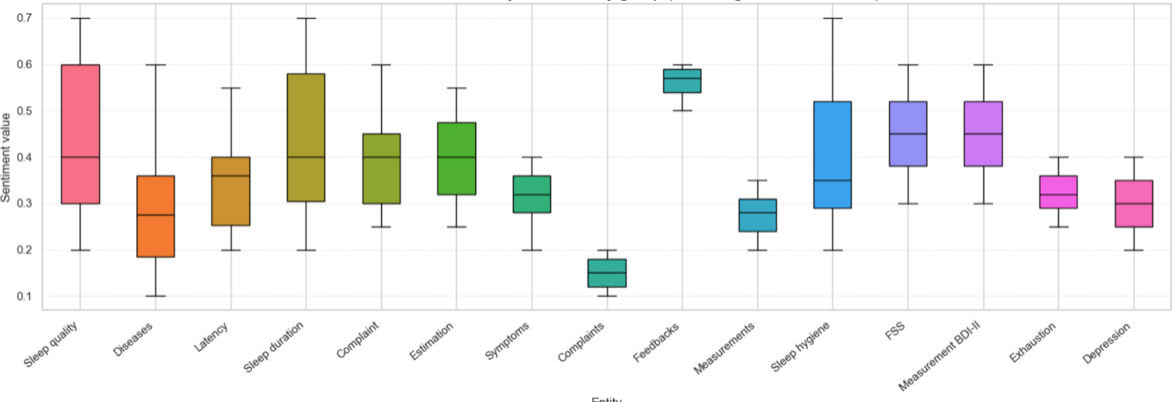
Sentiment distribution by clinical entity group. FSS: Fatigue Severity Scale; MSLT: Multiple Sleep Latency Test; MWT: Maintenance of Wakefulness Test.

**Figure 6.**
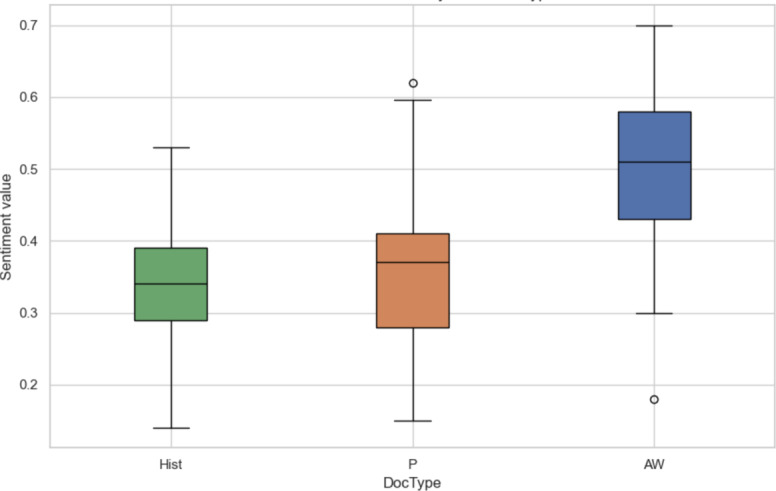
Sentiment distribution by document type (Hist:History, P:Polysomnography, P-AW:Actigraphy Wrist), 5-stage sentiment score normalized into value between 0 and 1.

**Figure 7.**
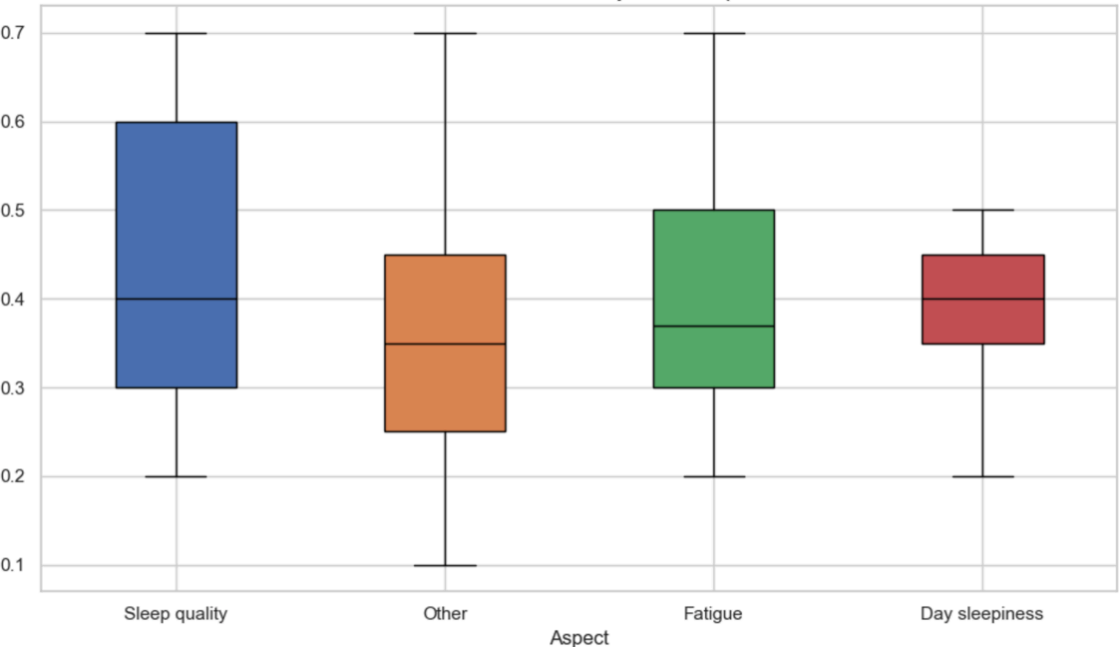
Sentiment distribution by sleep disorder aspect (quality, fatigue, and sleepiness).

Figures5^7^ visualize the distribution of sentiment values across different clinical contexts. Figure 5 presents sentiment grouped by clinical entity (eg, Sleep quality, Symptoms, and Feedback). Positive sentiment distributions were observed for Feedback and Sleep Hygiene, while negative sentiment dominated in entities such as Complaints and Diseases. Figure 6 shows sentiment variation across document types: patient-generated narratives (P) and Actigraphy Wrist (P-AW) reports tended to be more emotionally expressive than historical records (Hist). Figure 7 aggregates sentiment by clinical aspect groupings, showing that Sleep quality included more variability in sentiment, whereas Fatigue and Day sleepiness showed predominantly negative sentiment patterns—suggesting a greater emotional burden associated with these complaints.

#### Subjective-Objective Mismatch in Sleepiness Reporting

To examine potential misperceptions in day sleepiness, we compared subjective KSS reports with objective latency data from MSLT and MWT protocols. KSS values were normalized to a 0‐1 range using the formula: KSS_normalized=(9–Raw Score)/(9–1) This transformation enables alignment with the inverse latency scale.

**Figure 8.**
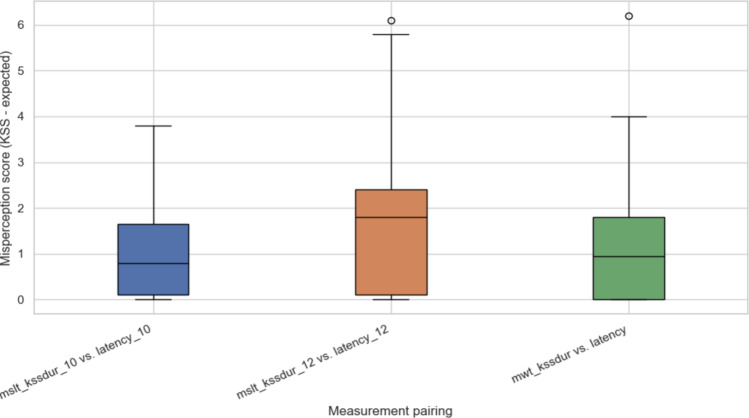
Mismatch between subjective sleepiness (Karolinska Sleepiness Scale) and objective sleep latency (Multiple Sleep Latency Test and Maintenance of Wakefulness Test) in day sleepiness documents with a threshold of 10 and 12 minutes of latency. KSS: Karolinska Sleepiness Scale; MSLT: Multiple Sleep Latency Test; MWT: Maintenance of Wakefulness Test.

Figure 8 presents the misperception score distribution across 3 test conditions: 10 minutes pair: mslt_kssdur_10 versus latency_10, 12 minutes pair: mslt_kssdur_12 versus latency_12, and mwt_kssdur versus latency. Misperception was defined as the deviation between normalized KSS and expected sleep latency levels. Across all pairings, positive misperception scores indicate that patients tend to report higher subjective sleepiness than measured objectively. The largest variability appeared in the mslt_kssdur_12 condition, while MWT tests showed narrower error bands.

Table 11 presents a representative subset of 10 patients illustrating questionnaire-expert-validated, text-predicted, and measured sleepiness scores. Although individual patterns vary, a general trend of overestimated sleepiness is observable.

**Table 11.** A snapshot and working example with 10 patients from the selected cohort of 100 patients for day sleepiness, illustrating normalized Karolinska Sleepiness Scale (momentary) and text-derived score, expert-labeled and predicted subjective scores, along with corresponding Multiple Sleep Latency Test latencies and normalized objective scores.

Patient ID	KSS^a^ stage	Text sentiment stage	MSLT^b^ latency (min)	Objective score (0‐4)	Differences KSS MSLT	Differences text MSLT
P001	2	2	5.04	2.992	−0.992	−0.992
P002	2	1	1.12	3.78	−1.78	−2.78
P003	3	2	7.76	2.45	0.55	−0.45
P004	0	1	1.13	3.77	−3.77	−2.77
P005	3	1	0.14	3.97	−0.97	−2.97
P006	2	1	15.99	0.80	1.20	0.20
P007	0	1	9.9	2.02	−2.02	−1.02
P008	3	1	10.54	1.89	1.11	−0.89
P009	3	3	8.83	2.23	0.77	0.77
P010	4	2	9.93	2.01	1.99	−0.01

a KSS: Karolinska Sleepiness Scale.

b MSLT: Multiple Sleep Latency Test.

A paired-sample *t* test conducted across the full cohort (n=100) revealed distinct patterns in the alignment between subjective and objective sleepiness assessments. While KSS scores showed a borderline nonsignificant divergence from MSLT-derived latencies (*t*_99_=2.456, *P*=.06), text-derived sentiment scores demonstrated a statistically significant misalignment (*t*_99_=2.324, *P*=.05). These findings suggest that free-text clinical narratives more consistently diverge from physiological measures of sleepiness than structured scales such as the KSS. This may reflect the emotionally nuanced and context-rich nature of patient-reported symptoms embedded in narrative records. Importantly, although both subjective measures showed overestimation of sleepiness, only the text-derived sentiment reached statistical significance (*P*=.05), indicating a consistent, though not necessarily larger, misalignment with physiological data. More details can be found in Multimedia Appendix 1.

## Discussion

### Principal Findings

This study introduced a sentiment analysis framework for clinical sleep narratives, revealing key insights into both method performance and clinical relevance. The BERT-BiLSTM architecture improved sentiment accuracy by addressing negations and drift, while the Mixtral 8X7B model outperformed Falcon 40B in aspect extraction due to better handling of complex German syntax. A fine-tuned GPT-2 model with reinforcement learning achieved high sentiment classification accuracy (87%) and offered a resource-efficient alternative to LLMs. Clinically, sentiment-derived scores revealed consistent misperception patterns—such as underestimated sleep latency and overestimated duration—highlighting the importance of aligning subjective and objective measures. These findings support the integration of sentiment-informed misperception analysis into sleep medicine workflows to improve diagnosis, treatment selection, and patient safety.

### Method Evaluation

In this study, we introduced and evaluated an aspect-based sentiment analysis approach tailored to clinical narratives in sleep medicine. Several key observations emerged from our findings. First, when mapping sentiment model outputs to scalar indicators of sleep disorders, it became necessary to adjust for negations and drifted sentiment—especially in baseline BERT models. Phrases that negated the presence of symptoms or diagnoses were frequently misinterpreted as negative sentiment. This issue was mitigated using a BERT-BiLSTM architecture, which improved performance through representation adaptation and fine-tuning.

For aspect (entity) extraction using prompted LLMs, recall was generally low, particularly for feedback-related content. In contrast, precision was high for identifying symptoms, diseases, and complaints. The poor recall is primarily attributed to the token limit and the generation style of Falcon 40B under one-shot prompting. Interestingly, few-shot prompting increased precision but further reduced recall and *F*_1_-score. This reveals a trade-off that may be addressed either by increasing computational capacity (current setup: 128 GB Nvidia DGX server) or optimizing prompting strategies.

The Mixtral 8X7B model significantly outperformed Falcon 40B, delivering higher precision and recall for aspect recognition, even under single-shot settings. Its ability to handle longer input contexts and complex linguistic structures—such as German postnegation—proved particularly advantageous in the clinical domain. These results support the use of Mixtral 8x7B for German-language medical NLP tasks where nuanced comprehension is critical.

Compared to BERT-BiLSTM, a fine-tuned GPT-2 model trained on 1200 sleep-related clinical sentences achieved comparable performance (accuracy: 81%). Incorporating keyword-guided SFT and 2 RL epochs with logit modification raised accuracy to 87%. This dual-phase tuning strategy enhanced the model’s flexibility and control. It also outperformed sequential prompting approaches commonly used with larger LLMs—offering higher precision with lower resource requirements, particularly important in privacy-sensitive clinical settings.

### Clinical Outcomes

The interpretation of sentiment-derived misperception patterns must consider both the nature of subjective reporting and the inherent limitations of objective measurement in clinical sleep data. Misperception—defined as a mismatch between how patients feel and how their physiological state is measured—varies across dimensions of sleep disorders, with important diagnostic and therapeutic implications.

A key finding in our study was the divergence in sentiment-derived scores for similar clinical constructs. For instance, patients frequently underestimated their sleep latency (eg, reporting quick sleep onset) yet simultaneously overestimated their sleep duration, contrary to PSG and MSLT results. This subjective-objective mismatch—commonly referred to as sleep misperception—was particularly evident across latency, duration, and fatigue indicators. While traditional statistical tests may not fully capture the nuances of sentiment-informed scores, the consistent directionality and magnitude of these mismatches highlight their clinical importance.

However, the objectification of sleep quality and fatigue is inherently complex. Short sleep latency, often seen as a marker of healthy sleep, can paradoxically indicate pathological hypersomnolence in conditions such as narcolepsy. Similarly, fatigue—unlike sleepiness—lacks a gold-standard physiological test and is primarily self-reported, making its objectification more elusive. These nuances emphasize the need to interpret subjective scores considering disorder-specific clinical contexts.

In contrast, daytime sleepiness offers a more quantifiable target for misperception analysis. Here, subjective ratings such as the KSS and text-derived sentiment scores can be directly compared with objective markers such as MSLT latency. The KSS is particularly useful in this context, as it captures state-based, momentary sleepiness, allowing for meaningful trial-by-trial comparisons. For example, KSS values close to 0 (indicating “extremely sleepy”) should theoretically align with MSLT latencies under 8 minutes. In our analysis framework, misperception magnitude increases when a patient reports low KSS (ie, “did not fall asleep”) while objective data confirm they did—for example, falling asleep within 10‐20 minutes in MSLT trials. This alignment enables fine-grained modeling of momentary misperception and supports targeted subanalysis of repeated MSLT and KSS pairs (eg, mslt_kssdur_10 vs mslt_sleeplatency_10).

From a clinical perspective, understanding the type and direction of misperception has actionable relevance.

For instance, in insomnia, patients often report exaggerated sleep difficulties (eg, longer sleep latency than measured). These cases may benefit more from cognitive behavioral therapy for insomnia rather than pharmacotherapy. Discussing the misperception with the patient is often therapeutic. However, because objective tests such as PSG are rarely used in routine insomnia management, wearable technologies (eg, smartwatches) could serve as surrogate tools to estimate sleep latency, duration, and fragmentation—offering scalable insight into real-world misperception.

In hypersomnia or sleep-disordered breathing, the opposite trend may occur—patients feel they slept well but exhibit abnormal sleep architecture or excessive daytime sleepiness in tests. In such cases, unrecognized sleepiness can elevate safety risks, such as motor vehicle or occupational accidents. Early identification of these cases using sentiment-objective comparison could trigger earlier clinical intervention or patient education.

Altogether, our sentiment analysis framework, enriched with structured objective metrics, offers a promising route for personalized assessment of sleep misperception. By comparing KSS, ESS, and text-derived sentiment scores against MSLT latency across multiple configurations, we can determine which input combination best captures the true subjective state. This multipronged analysis will inform future iterations of our clinical decision-support tools and help stratify patients for tailored treatment approaches.

#### Clinical Applications

Integrating sentiment analysis and misperception scoring into clinical workflows opens up new avenues for personalized, perception-aware sleep medicine:

1. Flag discrepancies in sleep latency and duration for targeted discussion or reassessment.
2. Stratify patients by degree of misperception to inform whether cognitive behavioral therapy for insomnia or pharmacotherapy is appropriate.
3. Highlight the underestimation of sleepiness in MSLT and MWT contexts for workplace or safety interventions.
4. Incorporate wearable data to validate or challenge patient self-reporting in follow-up care.

By aligning sentiment-derived patterns with objective sleep markers, clinicians can better understand not only what the patient reports but how they perceive their condition—a critical step toward improving diagnostic accuracy, treatment matching, and long-term outcomes.

### Conclusion and Future Work

This study demonstrates that sentiment analysis applied to structured clinical narratives can uncover meaningful patterns in how patients perceive and report sleep disorders—especially in identifying discrepancies between subjective self-reports and objective assessments. Among the models evaluated, the BERT-BiLSTM architecture showed strong performance in domain-specific sentiment detection. However, the fine-tuned German GPT-2 model, enhanced with supervised and reinforcement learning (PPO), achieved the highest accuracy for 5-stage sentiment classification, offering an effective balance between adaptability and computational efficiency.

Our findings highlight the clinical value of sentiment-based misperception modeling. For example, patients with insomnia often overestimate wakefulness, while those with hypersomnia or sleep apnea tend to underreport daytime sleepiness. The use of reinforcement learning techniques proved especially effective for capturing emotionally nuanced language in clinical narratives while maintaining sensitivity to domain-specific clinical distinctions.

However, this study is not without limitations. All data were drawn from a single clinical registry, which may limit the generalizability of findings across institutions or populations. Furthermore, the annotated training set for sentiment and entity extraction was relatively small, potentially constraining model robustness and the diversity of learned patterns.

In future work, we aim to expand the dataset by incorporating records from additional clinical centers and to enrich the annotation set to enable more nuanced model training and validation. Sentiment modeling will be extended to cover additional clinical entities such as pain and cognitive symptoms, while correlations between sentiment-derived misperception scores and treatment outcomes or resistance will be further explored. We also plan to integrate physiological features—such as microarousals and sleep stage transitions—to improve subtype classification and to leverage wearable sensor data to validate and calibrate sentiment-based misperception metrics. Ultimately, our goal is to develop a precision sleep medicine framework that integrates objective physiological markers with patients’ emotional and cognitive interpretations of their symptoms, captured through advanced sentiment and entity modeling of clinical narratives.

## Supplementary material

10.2196/70753 Multimedia Appendix 1 Mismatch between subjective sleepiness (Karolinska Sleepiness Scale) and objective sleep latency (Multiple Sleep Latency Test) with a threshold of 10 and 12 minutes among 100 patients.

## Abbreviations

AHI: Apnea-Hypopnea Index
ESS: Epworth Sleepiness Scale
FSS: Fatigue Severity Scale
*ICSD-III*: *International Classiﬁcation of Sleep Disorders, Third Edition*
KSS: Karolinska Sleepiness Scale
LLM: large language model
MSLT: Multiple Sleep Latency Test
MWT: Maintenance of Wakefulness Test
NLP: natural language processing
P-AW: Actigraphy Wrist
PPO: Proximal Policy Optimization
PSG: polysomnography
RL: reinforcement learning
SFT: supervised fine-tuning
SOL: sleep onset latency
TRL: Transformer Reinforcement Learning
TST: total sleep time

## References

[1] Michal M, Wiltink J, Kirschner Y (2014). Complaints of sleep disturbances are associated with cardiovascular disease: results from the Gutenberg Health Study. PLoS One.

[2] Kotterba S, Neusser T, Norenberg C (2018). Sleep quality, daytime sleepiness, fatigue, and quality of life in patients with multiple sclerosis treated with interferon beta-1b: results from a prospective observational cohort study. BMC Neurol.

[3] Mendonca F, Mostafa SS, Morgado-Dias F, Ravelo-Garcia AG, Penzel T (2019). A review of approaches for sleep quality analysis. IEEE Access.

[4] Zavecz Z, Nagy T, Galkó A, Nemeth D, Janacsek K (2020). The relationship between subjective sleep quality and cognitive performance in healthy young adults: evidence from three empirical studies. Sci Rep.

[5] O’Donnell D, Silva EJ, Münch M, Ronda JM, Wang W, Duffy JF (2009). Comparison of subjective and objective assessments of sleep in healthy older subjects without sleep complaints. J Sleep Res.

[6] Aurora RN, Caffo B, Crainiceanu C, Punjabi NM (2011). Correlating subjective and objective sleepiness: revisiting the association using survival analysis. Sleep.

[7] Stanyte A, Podlipskyte A, Alonderis A, Macijauskiene J, Burkauskas J, Steibliene V (2024). Relationship between subjective and objective fatigue and sleep characteristics in individuals with anxiety and mood disorders: an exploratory study. Physiol Behav.

[8] Valko PO, Hunziker S, Graf K, Werth E, Baumann CR (2021). Sleep-wake misperception. A comprehensive analysis of a large sleep lab cohort. Sleep Med.

[9] Abbey SE, Garfinkel PE (1991). Chronic fatigue syndrome and depression: cause, effect, or covariate. Rev Infect Dis.

[10] Hermans LWA, Leufkens TR, van Gilst MM (2019). Sleep EEG characteristics associated with sleep onset misperception. Sleep Med.

[11] Herzog R, Crosbie F, Aloulou A (2025). A continuous approach to explain insomnia and subjective-objective sleep discrepancy. Commun Biol.

[12] Devlin J, Chang MW, Lee K, Toutanova K BERT: pre-training of deep bidirectional transformers for language understanding. http://aclweb.org/anthology/N19-1.

[13] Behera RK, Jena M, Rath SK, Misra S (2021). Co-LSTM: Convolutional LSTM model for sentiment analysis in social big data. Inf Process Manag.

[14] Denecke K, Reichenpfader D (2023). Sentiment analysis of clinical narratives: a scoping review. J Biomed Inform.

[15] Kim Y (2014). Convolutional neural networks for sentence classification.

[16] Rehman AU, Malik AK, Raza B, Ali W (2019). A hybrid CNN-LSTM model for improving accuracy of movie reviews sentiment analysis. Multimed Tools Appl.

[17] Wang Z, Yang B Attention-based bidirectional long short-term memory networks for relation classification using knowledge distillation from BERT.

[18] Yang X, Li Y, Li Q, Liu D, Li T (2022). Temporal-spatial three-way granular computing for dynamic text sentiment classification. Inf Sci (Ny).

[19] Yu L, Dong J, Chen L (2021). PBCNN: packet bytes-based convolutional neural network for network intrusion detection. Comput Netw.

[20] Liu P, Qiu X, Huang X Recurrent neural network for text classification with multi-task learning.

[21] Ziegler DM, Stiennon N, Wu J (2020). FineTuning language models from human preferences. arXiv.

[22] Schulman J, Wolski F, Dhariwal P, Radford A, Klimov O (2017). Proximal policy optimization algorithms. arXiv.

[23] Pascual D, Egressy B, Meister C, Cotterell R, Wattenhofer R A plug-and-play method for controlled text generation.

[24] Denecke K, Deng Y (2015). Sentiment analysis in medical settings: new opportunities and challenges. Artif Intell Med.

[25] Aellen F, Van der Meer J, Dietmann A, Schmidt M, Bassetti CLA, Tzovara A (2022). The Bern Sleep Database: clustering of patients with sleep disorders. Sleep Med.

[26] Werra L Tune GPT-2 to generate controlled sentiment reviews. preprint, under review. Google Collab.

[27] Falcon 40B LLM repository. Hugging Face.

[28] Werra L, Belkada Y, Tunstall L TRL: transformer reinforcement learning. GitHub.

[29] Zhang H, Song H, Li S, Zhou M, Song D (2024). A survey of controllable text generation using transformer-based pre-trained language models. ACM Comput Surv.

[30] Mixtral 8x7b repository. Hugging Face.

[31] BERT base multilingual cased. Hugging Face.

[32] XLM-roberta base. Hugging Face.

[33] Werra L Tune GPT-2 to generate positive reviews. Hugging Face.

[34] German GPT-2.

[35] Code repository. GitHub.

