# Supplementary figures and images for: Analyzing Sleep Behavior Using BERT-BiLSTM and Fine-Tuned GPT-2 Sentiment Classification: Comparison Study
Source: JMIR Med Inform. 2025 Nov 10;13:e70753. doi: 10.2196/70753 (PMC12599995; doi:10.2196/70753)

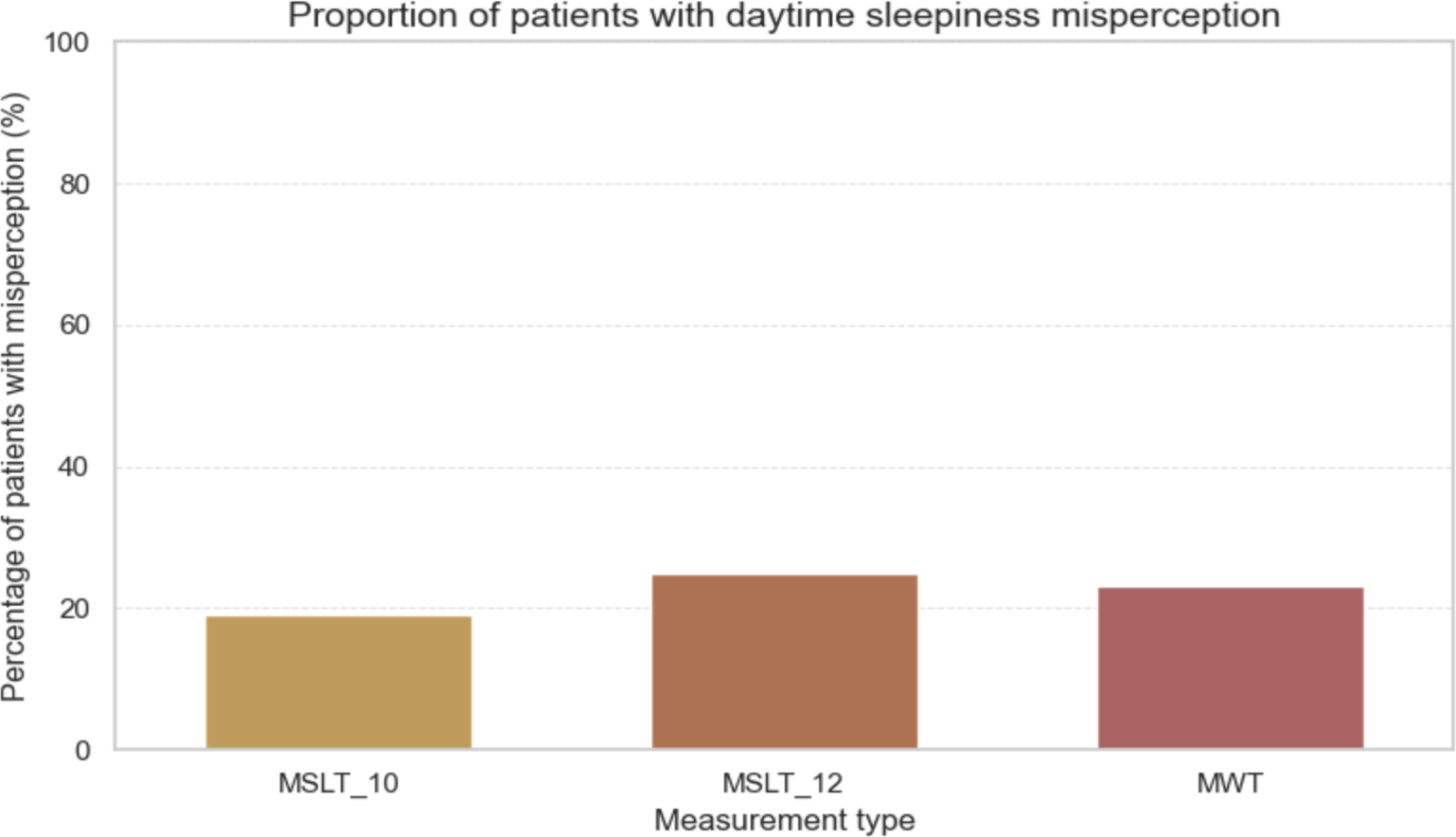

Supplement: Multimedia Appendix 1 [file medinform-v13-e70753-s001.png]
